# HIV self-testing in Spain: A valuable testing option for men-who-have-sex-with-men who have never tested for HIV

**DOI:** 10.1371/journal.pone.0210637

**Published:** 2019-02-13

**Authors:** Konstantinos Koutentakis, Juan Hoyos, María-Elena Rosales-Statkus, Juan-Miguel Guerras, Jose Pulido, Luis de la Fuente, María-José Belza

**Affiliations:** 1 Carlos III Health Institute, National Center of Epidemiology, Madrid, Spain; 2 Consortium for Biomedical Research in Epidemiology & Public Health (CIBERESP),Madrid, Spain; 3 Department of Public and Mother-child health, Faculty of Medicine, Madrid Complutense University, Madrid, Spain; 4 Carlos III Health Institute, National School of Health, Department of Health Programs, Madrid, Spain; 5 Carlos III Health Institute, National School of Health, Department of Epidemiology and Biostatistics, Madrid, Spain; Agencia de Salut Publica de Barcelona, SPAIN

## Abstract

**Background:**

We assessed the capacity of HIV self-testing to promote testing among untested men who have sex with men (MSM) and determined the most benefited subpopulations.

**Methods:**

An online questionnaire was disseminated on several gay websites in Spain from September 2012 to April 2013. We used Poisson regression to estimate factors associated with the intention to use self-testing if already available. Among those who reported intention of use, we assessed several aspects related to the testing and linkage to care process by type of barrier reported: low perceived risk (LR), structural barriers (SB) and fear of testing positive (FTP).

**Results:**

Of 2589 never-tested MSM, 83% would have used self-testing if already available. Intention of use was associated with age ≥30 (adj.PR, 95%CI: 1.05, 1.01–1.10), having had protected (adj.PR, 95%CI: 1.15, 1.02–1.30) or unprotected (adj.PR, 95%CI: 1.21, 1.07–1.37) anal intercourse and reporting FTP (adj.PR, 95%CI: 1.12, 1.05–1.20) or SB to access HIV testing (adj.PR, 95%CI: 1.23, 1.19–1.28). Among those who reported intention of using a self-testi, 78.3% declared it their preferred option (83.8% in the SB group; p<0.001), and 56.8% would always use this testing option (60.9% among the SB group; p = 0.001). In the case of obtaining a positive self-test, 69.3% would seek confirmatory testing, 15.3% would self-test again before taking any decision and 13.0% reported not being sure of what they would do.

**Conclusion:**

HIV self-testing in Spain has the potential of becoming a highly used testing methodology for untested MSM and could represent the gateway to testing especially among older, at risk MSM who report SB or FTP as main barriers to testing.

## Introduction

In Spain and in the rest of Western Europe, men who have sex with men (MSM) are the population most affected by HIV. Of the 1270 new HIV cases notified among MSM during 2016 39.4% were diagnosed at a late stage of infection (< 350mm^3^ CD4 count at diagnosis) [1]. From a public health perspective, to promote earlier diagnosis and to reduce the undiagnosed fraction of the epidemic is one of the main priorities in HIV prevention programs worldwide because treatment reduces infectiousness and contributes to stop onward transmission [2, 3].

Recommendations for MSM are that an HIV test should be performed at least every 12 months and every 3 when having unprotected sex with casual partners [4]. However, studies across Europe point out that these recommendations are far from being met [5–8]. In fact, the first European men who have sex with men internet survey (EMIS) found that the percentage of untested MSM in western European countries ranged from 16.2% in France to 37.2% in Finland [9].

In Spain, HIV testing is offered free of cost at all levels of the public health system. It is also performed in a network of sexual health clinics and units that not only offer it free of cost but also anonymously. Confidential and anonymous rapid testing is also conducted for free by a number of community based organisations (CBO) and, in 5 regions, there are also pharmacy based rapid testing programmes where those who wish, can get tested by a pharmacist. In spite of the variety of settings, 26.1% of MSM in Spain have never been tested for HIV [10].

Barriers to testing include reasons related to risk perception, fear of stigma and discrimination and to health consequences derived from a positive result [11]. Additionally, there are also a number of structural barriers related to the characteristics of the diagnostic settings that include lack of anonymity and or/ confidentiality, having to wait, having to return to receive the result of a test, and the need to discuss sexual behaviours [11].

In this sense, self-testing is a methodology that could help to alleviate some of the aforementioned barriers. First approved in the US in 2012, self-testing has slowly but steadily spread across high-income countries. At present it is being marketed in the United Kingdom (UK), France, Belgium and Italy. In Spain self-testing kits started to be commercialized in January 2018 [12]. Self-testing provides individuals with the opportunity of learning their serostatus conveniently since it requires no appointments, no queues, no time invested in getting to testing sites and no pre and post-test discussion. On the other hand it also raises some concerns. The lack of face to face pre-test contact with a professional is one of the most common ones. Thus, it is important to provide good written and online materials to inform potential users both about the benefits and limitations (i.e window period and the need of a confirmation test) of self-testing. Coping with a positive self-test alone is another concern and support mechanisms need to be put into place to offer support and information for those in this situation. Finally there are also concerns about users who obtain a reactive self-test and do not seek confirmation testing and subsequent linkage to specialist care [13–16].

If self-testing is capable of eliminating some of the known barriers and of facilitating access to HIV testing among those who remain untested, it is crucial to know which subpopulations would use it. The literature assessing the impact that the introduction of self-testing could have in untested MSM is very limited and mostly comes from Australia and the US. In Europe, we found very few studies assessing this aspect in this population [17–19] and none of them focus on never tested individuals. Furthermore, we do not know what type of barriers self-testing would help to remove and what would happen in the case of receiving a positive result. Acts following a reactive self-test and preferences regarding confirmation sites remain unknown and are important elements that need to be taken into account for the future development of the strategy.

In the present paper we assess the impact that self-testing could have among untested MSM in Spain. We identify the subgroups that would benefit the most from its introduction and assess self-testing’s capacity of overcoming barriers that deter people from testing. Additionally we also investigate several key aspects related to the testing process and linkage to care that need to be considered to assure an effective introduction of this methodology.

## Methods

### Study participants

Inclusion criteria were being > = 18 years of age and having reported lifetimes sex with other men. Of the 9489 participants who met the inclusion criteria and self-identified as HIV negative or had an unknown serostatus, we selected 2818 MSM who reported no previous HIV testing experience. Additionally, we excluded 229 who did not answer the main outcome variable (would have used self-testing if already available). Thus, 2589 participants were included in the final analysis. No incentives were given for participation.

### Study procedures and measurements

We conducted an internet based survey between September 2012 and April 2013. Our study was advertised mainly in gay dating websites operating in Spain. Details about the study procedures have been reported elsewhere [20]. Briefly, those who chose to access the survey website were directed to an informative screen. Here, participants learned about the purpose of the study and the estimated time to complete the questionnaire (15 min). They were also informed about the fact that no Internet Protocols or cookies were collected and that data was collected confidentially and anonymously. The questions were designed to elicit no identifying information. Thus, participants were asked to report their age in years rather than their date of birth. Likewise, we did not include questions on the city or town of residence; instead, we asked only about the approximate number of inhabitants. Given the online nature of the study, written consent was obtained by an “I agree to participate” button which was presented to participants at the end of the informative screen. Once they clicked, they accessed the questionnaire. The study and all its materials (including the questionnaire) were approved by the Carlos III health institute ethical committee (CEI PI 70_2015). All procedures performed in studies involving human participants were in accordance with ethical standards of the institutional research committee and with the 1964 Helsinki declaration and its later amendments or comparable ethical standards. Study did not include minors.

The questionnaire was designed in Spanish and collected data on socio-demography, lifestyle, sexual behaviours, testing history and several aspects related to HIV self-testing. The main outcome for this analysis was intention to use self-testing which was assessed with the question “Would you have used a self-test for HIV, had it been available in pharmacies in Spain?”. Replies initially were set as “yes”, “probably yes”, “not sure”, “probably no”, “no” and were transformed into a binary variable for analysis: “yes/probably yes” and “I am not sure/probably no/no”.

To assess the barriers of access to HIV testing we used a question with eleven closed response options and one open-ended. Participants were asked to choose their single most important reason. For the analysis, they were recoded into a four-category variable, as follows:

- Low perceived risk for HIV, which included the options: “I found myself very healthy”, “I thought that with my behaviours I could not be infected”.
- Fears of testing positive, which included: “Fear of the consequences for my health”, “Fear of not being able to get / lose my job”, “Fear of not being able to get/lose a residence permit”, “Fear of rejection or discrimination”.
- Structural barriers, including the options: “I didn't want to go to my GP/health center”, “Concerns arising from the loss of anonymity while being tested”, “I knew I had to wait several days to get the results”, “I wanted to do it in a private centre but had no money”, “Feel discomfort answering intimate questions”
- “Other reason”: To allow participants to express another reason not listed among the aforementioned.

The questions of the survey used for this analysis can be found as supporting information (S1 Appendix).

### Statistical analysis

First, we performed a descriptive analysis of the main characteristics of never tested MSM.

Second, we calculated the proportion of participants that reported having intentions of using a self-test if already available and cross-tabulated our data by relevant sociodemographic and behavioral variables as well as by type of self-reported barrier for testing.

To address associations between intention to use self-testing and MSM characteristics, we calculated crude and adjusted prevalence ratios (PRs) and their corresponding 95% confidence intervals (CIs). We chose to use poisson regression with robust variance because it is a better alternative than logistic regression in cross-sectional studies with frequent outcomes [21, 22]. All the statistically significant factors at p<0.2 in the single variable analysis were introduced a multivariable regression model. We used the minimum Akaike information criteria (AIC) values to perform model comparisons and select the optimal model. Respondents with missing data on > = 1 variables of the multivariable model, were not included in the regression model.

We performed the analysis using STATA 11 software (StataCorp, Texas, United States, United States).

A third level of analysis, focused on those who reported intention to use a self-test. In this group, we assessed the preference towards self-testing among all testing options, the type of self-test preferred (blood/oral) and whether they would use it alone or accompanied by someone. We also assessed the number of times they would have used a self-test, in what way they would incorporate it to their usual testing pattern, and what would be their preferred setting to seek for a confirmation test if the self-test was reactive. This analysis has been stratified by the type of barrier self-reported by participants and the relationship between the categorical variables was assessed using Pearson’s Chi-square test. Statistical significance was defined as a two-tailed p value of less than 0.05.

## Results

Some 91.8% of all participants were born in Spain, 44.8% were 30 years of age or older and 46.7% had finished a university degree. In the preceding 12 months, most had lived in Spain (96.0%) in a city of more > = 100.000 inhabitants (58.1%). Additionally, 41.3% reported having had sex with men and women, 20.3% had kept their sex life with other men in total secrecy and 60.2% were not related to the gay scene (Table 1).

**Table 1:** Prevalence and factors associated with intention to use self-testing if already available among never tested MSM in Spain (N = 2,589^a^).

	Total (N = 2589)		Participants with intentions to use self-testing (N = 2161)						
	N	% (col)	n	% (row)	cPR	95% CIs	P	aPR	95% CIs
**Age**									
<30 years old	1428	55.2	1160	81.2	1.00			1.00	
≥30 years old	1158	44.8	998	86.2	**1.06**	**1.03–1.10**	**0.001**	**1.05**	**1.01–1.10**
**Education level**									
<University	1376	53.3	1159	84.2					
≥University	1206	46.7	997	82.7	0.98	0.95–1.02	0.288		
**Place of recidence**^**b**^									
Latin-America	23	1.0	21	91.3	1.00			1.00	
Other European country	64	2.7	50	78.1	0.86	0.71–1.03	0.091	0.86	0.70–1.05
Spain	2310	96.0	1938	83.9	0.92	0.81–1.04	0.193	0.93	0.79–1.08
Other Country	10	0.4	10	100.0	1.01	0.97–1.24	0.158	1.13	0.95–1.34
**Settlement size (inhabitants)**^**b**^									
>100,000	1389	58.1	1156	83.2	1.00			1.00	
<100,000 (within 30 km of a big city)	619	25.9	518	83.7	1.01	0.96–1.05	0.798	1.01	0.97–1.05
<100,000 (> = 30 km of a big city)	383	16.0	330	86.2	1.04	0.99–1.09	0.145	1.01	0.97–1.06
**Cohabitation**^**b**^									
Lives alone	830	34.7	712	85.8	1.00			1.00	
Lives with sexual partner	484	20.2	418	86.4	1.01	0.96–1.05	0.769	0.99	0.95–1.04
Lives with others (not a sexual partner)	1081	45.1	880	81.4	**0.95**	**0.91–0.99**	**0.010**	0.98	0.94–1.02
**Sexual partners (lifetime)**									
Men & women	1070	41.3	898	83.9	1.00				
Men only	1519	58.7	1263	83.1	0.99	0.96–1.03	0.598		
**Disclosure of sexual orientation**									
Has not disclosed his sexual orientation	492	20.3	403	81.9	1.00			1.00	
Has disclosed his sexual orientation to someone	1937	79.7	1639	84.6	1.03	0.99–1.08	0.163	1.03	0.98–1.08
**Relation with gay culture**									
Member of a gay CBO^c^	59	2.5	42	71.2	1.00			1.00	
Not member of gay CBO but frequents the gay scene	875	37.3	755	86.3	**1.21**	**1.03–1.43**	**0.022**	1.08	0.93–1.27
Not related to gay scene	1410	60.2	1176	83.4	1.17	0.99–1.38	0.058	1.06	0.90–1.23
**Anal intercourse & condom use**^**b**^									
No anal intercourse	129	5.3	86	66.7	1.00			1.00	
Had, but all were protected^d^	937	38.7	759	81.0	**1.22**	**1.07–1.38**	**0.002**	**1.15**	**1.02–1.30**
Had unprotected anal intercourse	1357	56.0	1190	87.7	**1.32**	**1.16–1.49**	**0.001**	**1.21**	**1.07–1.37**
**Has been paid for sex**									
Yes	99	4.1	84	84.8	1.00				
No	2340	95.9	1963	83.9	0.99	0.91–1.08	0.793		
**Has paid for sex**									
Yes	176	7.2	150	85.2	1.00				
No	2266	92.8	1900	83.8	0.98	0.92–1.05	0.618		
**Injection drug use**	2442								
Never	2384	98.9	2002	84.0	1.00				
Yes, > = 12 months ago	15	0.6	11	73.3	0.87	0.64–1.19	0.385		
Yes, in the last 12 months	12	0.5	10	83.3	0.99	0.77–1.28	0.953		
**History of STIs**									
No	1798	74.9	1491	82.9	1.00			1.00	
Lifetime diagnosis of STI	603	25.1	523	86.7	**1.05**	**1.01–1.09**	**0.018**	0.99	0.96–1.03
**Main reason for not HIV testing**									
Low perceived risk for HIV	1236	48.2	936	75.7	1.00			1.00	
Fear of testing positive	229	8.9	193	84.3	**1.11**	**1.04–1.19**	**0.001**	**1.12**	**1.05–1.20**
Structural barriers	931	36.3	884	94.9	**1.25**	**1.21–1.30**	**0.000**	**1.23**	**1.19–1.28**
Other	166	6.5	122	73.5	0.97	0.88–1.07	0.544	0.98	0.89–1.08

cPR: crude prevalence ratio; aPR: adjusted prevalence ratio; CI: confidence interval

^a^ Due to missing data the numbers might not add up to totals

^b^ In the last 12 months

^c^ Community-Based Organization

^d^ Protection referred to condom use only

More than half (56.0%) had had at least one unprotected anal intercourse (UAI) in the last 12 months, 7.2% had ever paid for sex, 4.1% had ever been paid for sex and 25.1% reported having been diagnosed with a sexually transmitted infection (STI). Very few (1,1%) had ever injected drugs (Table 1).

Regarding the reasons for never having been tested before, 48.2% of the participants reported low perceived risk which included the following reasons “I thought that with my behaviors I could not be infected “(37.3%) and “I found myself very healthy” (10.5%). Structural barriers were reported by 36.4% and included: “Concerns arising from the loss of anonymity” (16.5%), “Feeling discomfort answering intimate questions” (8.3%), “I didn't want to go to my GP/health center” (7.7%), “I knew I had to wait several days to get the results” (1.9%), “I wanted to do it in a private center but had no money” (1.5%). Fears derived from testing positive were reported by 8.9% of the participants and included “Fear of the consequences for my health” (6.1%),“Fear of rejection or discrimination” (2.1%), “Fear of not being able to get/lose work” (0.5%) and “Fear of not being able to get/lose residence permit” (0.1%). The remaining 6.4% of the participants reported “other reasons”.

The overall proportion of participants who reported intentions of using a self-test if already available, was of 83.5% (95%CI: 82.0–84.9): 53.3% answered “yes” and 30.2% “probably yes”.

The results of the poisson regression analysis to assess the association of lifestyle and behavioral characteristics with the intention to use self-testing are shown in Table 1. In the multivariate model, variables that remained significantly associated with intention to use self-testing were age of > = 30 years (PR, 95%CI: 1.05, 1.01–1.10), having had anal intercourse in the last twelve months either protected or not (PR, 95%CI: 1.15, 1.02–1.30; PR, 95%CI: 1.21, 1.07–1.37; respectively), reporting fears derived of testing positive (PR, 95%CI: 1.12, 1.05–1.20) and structural barriers to get tested for HIV (PR, 95%CI: 1.23, 1.19–1.28 (Table 1). The proportion of missing data of the independent variables are included as supplementary material (S1 Table)

The proportion of participants that identified self-testing as their preferred testing option was of 78.3% and was higher among those who reported structural barriers (83.8%) than among those who were never testers because of low risk perception (74.5%) or fear related reasons (71.0%) (p<0.001). Almost half (45.9%) reported that they would be willing to use any of the two self-testing options (blood or saliva) and 84.5% that they would prefer to use it alone instead of with someone (Table 2).

**Table 2:** Type of self-test preferred, aspects of the HIV self-testing process and potential use by type of barrier reported by those who expressed intention to use self-testing if already available.

	Type of barrier								
	Low risk perception		Fear		Structural barriers		Total		P
***N*, *% (row)***	936	46.5	193	9.6	884	43.9	2013	100.0	
**Refers self-testing as the preferred testing option**	697	74.5	137	71.0	741	83.8	1575	78.3	<0.001
**Type of self-test preferred**									0.60
Blood	128	13.9	30	15.9	129	14.8	287	14.5	
Saliva	383	41.5	71	37.6	331	38.0	785	39.6	
Either	412	44.6	88	46.6	411	47.2	911	45.9	
**How would you prefer to use a self-test kit?**									0.64
Alone	785	84.7	157	82.2	742	84.9	1684	84.5	
Acompanied by someone	142	15.3	34	17.8	132	15.1	308	15.5	
**Times a self-test would have been used if already available**									<0.001
Only once	482	51.8	56	29.0	317	36.2	855	42.8	
Two to trhee times	357	38.3	100	51.8	403	46.0	860	43.0	
Four or more times	92	9.9	37	19.2	156	17.8	285	14.2	
**Frequency of use if self-test kits were available**									0.013
I would never/rarely use it, I rather get tested by a trained professional	129	14.3	30	16.1	93	10.9	252	13.0	
It would be my usual testing method, although I would also seek testing by trained professionals	293	32.4	54	29.0	240	28.2	587	30.2	
I would always use it	482	53.3	102	54.8	519	60.9	1103	56.8	
**Reaction following a positive self-test**									<0.001
Not sure what would I do	89	9.6	49	25.5	122	14.0	260	13.0	
I would self-test again before taking a decision	132	14.2	27	14.1	146	16.7	305	15.3	
I would seek for confirmation in a sexual health clinic	276	29.6	53	27.6	264	30.2	593	29.7	
I would seek for confirmation in primary care	316	33.9	42	21.9	241	27.6	599	30.0	
I would seek for confirmation in a private laboratory	62	6.7	10	5.2	54	6.2	126	6.3	
I would seek for confirmation in a CBO/NGO	39	3.1	8	4.2	29	3.3	66	3.3	
I would seek for confirmation in a family planning service, pharmacy or others	27	2.9	3	1.6	18	2.1	48	2.4	

Participants who reported fear and structural related barriers tended to think they would have used it more times (p<0.001) than those who reported low risk perception or fear. If approved, self-testing would always be used by 56.8% of the participants and those who reported structural barriers chose this option more frequently (60.9%) than the other two groups (p = 0.013) (Table 2).

In the case of receiving a positive result, the preferred confirmation sites would be primary care (30.0%) and sexual health clinics (29.7%), although a substantial percentage of MSM with fear related barriers (25.2%) reported not being sure of what would they do (p<0.001) (Table 2).

## Discussion

A large majority of the untested MSM we recruited online reported that they would have used an HIV self-test if already available. Intention to use was especially high among older MSM who reported anal intercourse in the las 12 months and among those who reported barriers to testing not related to risk perception. Among those who expressed willingness to self-test, a large majority identified it as their preferred mode of testing and reported that, if available, they would use this method exclusively. Most would attend primary care and sexual health clinics for confirmatory purposes although a quarter of those expressing fear of the consequences of a positive result are not sure of what would they do if they obtained a positive result.

The percentage of untested MSM was slightly higher than that reported by a Spanish study that also analyzed MSM recruited online (26%) [10]. We found three European studies that assessed the willingness of MSM to use HIV self-testing kits [17–19] and proportions ranged from 54.6% [19] to figures of nearly 90% and, therefore, very similar to our results [17, 18]. Of these studies, only two assessed the association between testing history and willingness to use a self-test kit [18, 19] and they present conflicting results. Whereas in the study by Greacen et al. having never tested before was associated with interest in accessing an HIV test, in the study by Rosales et al. it was the opposite and those with previous testing experience were the ones that reported higher levels of interest in purchasing a self-test. Nevertheless, both of them include testing history as an independent variable making it impossible to assess which particular sub-groups of never testers would benefit from its approval. As far as we know, the present paper represents the first study at a European level that tries to distinguish which groups of never-testers would use it if approved.

In this sense, the fact that older age is associated with willingness to use a self-test is an important issue as they are more affected by late diagnosis in Spain [1] and elsewhere in Europe [23]. It also means that it would benefit never tested MSM with longer periods at risk of HIV acquisition and, hence, a subgroup with higher number of unknown infections as suggested in several studies [24, 25]. The fact that participants involved in UAI in the last 12 months were the ones who reported higher willingness of using a self-test kit stresses the potential of self-testing as a tool that could play an important role on promoting testing among a hard to reach population who is not using pre-existing testing options in spite of being at risk of acquiring HIV.

A key factor that has not been assessed until now is what type of barriers HIV self-testing would help to remove. Populations with different barriers will be in the need of different testing strategies. In this sense, according to our results, the group less benefited by the approval of HIV self-testing kits would be those MSM that reported low risk perception. Low risk perception affects the intention of actively seeking an HIV test and those reporting it could probably be better targeted by provider initiated strategies [26] and tailored interventions to increase their risk perception.

Self-testing kits might be better fit for those who are not willing to access existing services due to structural barriers or because of fears derived from a potential positive result. This is in line with our results. Those facing structural barriers are a relevant group in size in our sample and represent almost 4 in 10 of the untested MSM. In Spain, where testing services are provided in a large variety of contexts, structural barriers could have to do more with the acceptability rather than with the availability of services. In this sense, some characteristics of self-testing such as complete anonymity, not having to deal with the anxiety of long periods of wait for the test results, convenience, or not having to discuss one’s sex life with a health provider, might be some of the reasons that make this testing option such a valued option for untested MSM who reported structural barriers. Never tested participants affected by barriers related to fears derived from a positive result are a much smaller group in size but would also benefit greatly from the introduction of self-testing kits.

According to our results, self-testing could become a very popular option among untested MSM. Most participants reported that it would become their favorite testing option and more than half said that they would only use this option. This however represents a major challenge: providing HIV confirmation testing and linkage to care services for all those who obtain a positive self-test. In this sense, further studies should be carried out to monitor confirmation testing and linkage to care among individuals receiving a positive self-test as data on the subject are extremely scarce at the moment [27, 28]. Most of our participants reported that they would seek for confirmation in the case of obtaining a positive self-test but, especially among those who faced fear related barriers, there is a substantial group that reported not being sure of what they would do in this scenario. It is important to ensure that in the case of obtaining a positive result a confirmatory test is performed within a time period that allows fulfilling the requirements of optimal linkage to care.

Several limitations need to be considered when interpreting these results. Although it is true that online dating websites are an increasingly frequent way of socializing and meeting new sex partners, the generalisation of these results to the overall untested MSM population needs to be made with caution. The data analysed in this paper, was collected immediately after the approval of self-testing in the US. Since then, self-testing has also been approved and legalised in several other European countries. MSM could have become more knowledgeable about it and some current opinions could be different to those from back in 2012–2013. Additionally, we do not know how answers based on the hypothetical situation of self-testing already being marketed, can translate into actual use. Those who expressed intentions of using it, could be reflecting a preference or sympathy towards this option. This however, does not mean it would necessarily lead to the purchase and use of a self-test kit when made available.

The proportion of missing values among those who reported medium or low testing intentions tended to be slightly higher than among those with high testing intentions (S1 Table). This needs to be taken into account when interpreting the results as it could be leading to an overestimation of those reporting high testing intentions. Nevertheless, its influence should be minimal given that the proportion of missing data is always under the 10% mark (0.1%- 9.5%). The difference of missing value rates should not change the interpretation of the results of our main outcome variable which is very frequent (83.5%).

The high acceptability of this testing method underlines its capacity of being a potentially relevant tool to promote testing among a severely undertested population. It would especially benefit subpopulations of older age, that have been involved in unprotected anal sex and that do not want to access already existing services due to structural barriers or because of fear derived from a positive result. Its high potential impact also represents a major challenge to ensure clear confirmation pathways to enable swift and effective linkage to care for those who obtain a positive self-test result.

## Supporting information

S1 Appendix Survey questions used in this study. (DOCX)

S1 Table Percentage of missing values in variables included in the final multivariable model by testing intention (N = 2589) *fisher's exact test. (DOCX)

S1 Dataset Data used in the analysis. (XLSX)
