# Supplementary material for: HIV self-testing in Spain: A valuable testing option for men-who-have-sex-with-men who have never tested for HIV
Source: PLoS One. 2019 Feb 13;14(2):e0210637. doi: 10.1371/journal.pone.0210637 (PMC6373894; doi:10.1371/journal.pone.0210637)
Supplement: S1 Appendix — (DOCX) [file pone.0210637.s001.docx]

**S1 Appendix. Survey questions used in this study**

**Intentions to use a self-test**

**Imagina que hace tiempo que el AUTOTEST se hubiera podido comprar en farmacia, ¿crees que ya te lo hubieras hecho tú mismo en algún momento?**

 Seguro que SÍ

Probablemente SÍ

No estoy seguro

Probablemente NO

Seguro que NO

**Would you have used an HIV self-test , had it already been available in pharmacies?**

Yes

Probably yes

Not sure

Probably no

No

**Age**

**¿Cuántos años tienes?** ________

**¿How old are you?** ________

**Place of birth**

**¿Has nacido en España?**

Sí

No, en otro país 🡪 ¿Cual?

**Were you born in Spain?**

Yes

No, in a diffferent country 🡪 Which one?

**Educational level**

**¿Cuál es el máximo nivel de estudios que has completado?**

Ninguno o estudios primarios (hasta los 12 años)

Enseñanza secundaria de primera etapa o formación profesional de grado medio (el nivel que debe acabarse a los 16 años)

Enseñanza secundaria de segunda etapa o formación profesional de grado superior (el nivel que debe acabarse a los 18 años)

Estudios universitarios

Otros/no sé clasificarme: describe con detalle los estudios más altos que has acabado:

**Which is the highest educational level you have completed?**

None or primary education (education up to 12 years of age)

First level secondary or middle grade vocational training (the level that should be finished at 16 years of age)

Second level secondary or upper grade vocational training (the level that should be finished at 18 years of age)

University studies

Others: please specify

**Place of residence**

**¿En los últimos 12 meses, donde has video la mayor parte de este tiempo?**

En España 🡪 ¿En qué Comunidad Autónoma?

En otro país 🡪 ¿Cuál?

**Where have you lived during most of the last 12 months?**

In Spain🡪 In which autonomous region?

In another country🡪 Which?

**Settlement Size**

**¿Cuántos habitantes tiene la localidad en que has vivido en los últimos 12 meses**

**(aproximadamente)?**

Más de un millón

Entre 500 mil y un millón

Entre 100 mil y 500 mil

Entre 50 mil y 100 mil

Entre 10 mil y 50 mil

Menos de 10 mil

**How many inhabitants does the place you have lived most of the last 12 months have (approximately)?**

More tan 1.000.000

Between 500.000 and 1.000.000

Between 100.000 and 500.000

Between 50.000 and 100.000

Between 10.000 and 50.000

Under 10.000

**Cohabitation**

**En los ÚLTIMOS 12 MESES la mayor parte del tiempo has vivido:**

Solo

Con otras personas

**¿Con quiénes? Señala TODAS las opciones que correspondan**

Cónyuge/pareja masculina

Cónyuge/pareja femenina

Hijos/as (propios, adoptados o de la pareja)

Padre/madre/tutor, suegro/a u otros familiares carnales o políticos

Amigos/compañeros de piso

Otras personas. Especifícalas:

**In the last 12 months; With whom have you lived most of the time?**

Alone

With other people

**With whom? Please select ALL the options that apply**

Male partner

Female partner

Son(s)/daughter(s) (own, adopted, or partners’)

Father/mother/tutor/father or mother in law or other family members

Friends/flatmates

Other persons: please specify

**Sexual partners (lifetime)**

**El término "relaciones sexuales" puede aplicarse a conceptos diversos. Aquí consideramos SÓLO aquellas en las que hay penetración vaginal, anal u oral.**

**Teniendo en cuenta la definición anterior a lo largo de tu vida ¿Con quién has tenido relaciones sexuales?**

Nunca he tenido relaciones sexuales, ni con hombres ni con mujeres

Sólo con mujeres

Más a menudo con mujeres, pero al menos una vez con un hombre

Igual con hombres que con mujeres

Más a menudo con hombres, pero al menos una vez con una mujer

Sólo con hombres

**The term “sexual relations” can apply to different concepts. Here, we consider ONLY those with vaginal, anal or oral penetration**

**According to this definition ¿With whom have you ever had sexual relations?**

I have never had sex neither with men or women

Only with women

More often with women but at least once with another man

Equally with men and women

More often with men but at least one time with a woman

Only with men

**Relation with gay culture**

**Dinos cuáles de las siguientes situaciones describen tu relación con la cultura y el ambiente gay.**

Señala TODAS las opciones que correspondan

Soy miembro o colaboro con una asociación gay

Suelo salir por locales de ambiente gay con amigos/as

Sólo acudo a locales de ambiente gay para ligar

Casi nunca he estado en locales de ambiente gay

**Which of the following situations best ddescribes your rleationship with the gay scene and culture.**

I am a member or collaborate with a gay association

I usually hang out in gay venues with my friends

I only go to gay venues to hook-up with someone

I’ve hardly been to gay venues.

**Unprotected sex**

**¿Y aproximadamente con cuántos tuviste relaciones ANALES SIN preservativo en este periodo? (en los últimos 12 meses)**

Con ninguno

Uno

Dos

3-4

5-9

10-19

20-50

Más de 50

**And with how many did you have unprotected anal intercourse? (in the last 12 months)**

None

One

Two

3-4

5-9

10-19

20-50

Over 50

**Intravenous drug use**

**¿Te has inyectado drogas alguna vez?**

Sí, pero hace más de 12 meses

Sí, en los últimos 12 meses

No, nunca

**Have you ever injected drugs?**

Yes, more than 12 months ago

Yes, in the last 12 months

No never.

**STI diagnosis**

**¿Alguna vez te han diagnosticado una enfermedad de transmisión sexual? Ejs: gonococia, sífilis, clamidia, herpes genital, tricomonas, etc.**

Sí

No

**¿Cuánto tiempo hace que tuviste esta última enfermedad?**

Menos de 12 meses

Hace más de un año pero menos de 5

Hace 5 años o más

**Have you ever been diagnosed with a sexually transmitted infection such as gonococci, syphilis, genital herpes, chlamydia, trichomonas etc.?**

Yes

No

**When was the last one diagnosed?**

Less than 12 months ago

Between 1 and 5 years ago

5 or more years ago

**Transactional sex**

**Durante los ÚLTIMOS 12 MESES, ¿has pagado para tener relaciones sexuales?**

Sí

No

**Y ¿te han pagado por tenerlas?**

Sí

No

**In the LAST 12 MONTHS, have you been paid in exchange of sex?**

Yes

No

**And, have you paid for sex?**

Yes

No

**Main reason for not testing for HIV**

**¿Cuál fue la razón principal para no habértela hecho antes?**

Me encontraba muy sano

Pensé que con los comportamientos que había tenido no podía estar infectado

Tenía miedo a las consecuencias para mi salud

Tenía miedo a perder el trabajo o que me impidiera encontrar otros

Estaba sin permiso de residencia o trabajo y pensaba que tendría problemas para conseguirlo

Tenía miedo al rechazo o discriminación

No quería ir a mi médico de cabecera / centro de salud

No sabía dónde acudir para hacérmela sin que me conocieran, sin tener que identificarme

Sabía que tendría que esperar bastantes días para conocer el resultado

Quería hacérmela en un centro privado y no tenía dinero para pagarla

Me incomodaba la idea de tener que responder a preguntas íntimas y personales

Otras. Especificar:

**What is the MAIN reason for not having been tested before?**

I feel very healthy

I think that, with my behaviours, I cannot be infected

I am afraid of the consequences for my health.

I am afraid I might lose my job or not find one if the test is positive.

I do not have a residence or work permit, and I think that I might have problems to obtain one if the test is positive.

I have fear of rejection or discrimination.

I don't want to go to my family doctor/ health centre.

I don't know where to go to get tested anonymously or where no one can recognize me.

Having to wait several days to know the result stresses me out

I want to get tested in a private centre but I cannot afford it.

I feel discomfort answering intimate and personal questions.

Others [Specify].

**Preference for self testing**

**Imagina que decides hacerte la prueba del VIH. De los siguiente servicios,¿Cuál preferirías para hacértela?**

El médico de cabecera

Un centro médico anónimo de diagnóstico del VIH y enfermedades de transmisión sexual

Un servicio de urgencias de un hospital

ONGs de sida o drogas (en su sede o en unidad móvil)

Una farmacia

Tú mismo, en casa (como la del embarazo)

**Imagine you decide to test for HIV: Which of the following settings would you prefer?**

Family doctor

An anonymous medical center for the diagnosis of HIV and other STIs

An Emergency department

An NGO (at their office or in a mobile unit)

A Pharmacy

On your own, at home (like a pregnancy test)

**Times a self-test would have been used if already available**

**¿Cuántas veces crees que lo hubieras empleado?**

Sólo una vez

Dos o tres veces

4-5 veces

Más de 5 veces

**How many time do you think you would have used it?**

Only one time

Two or three times

Four or five times

More than 5 times

**Reaction following a positive self-test**

**Si te hicieras el AUTOTEST y el resultado fuera positivo, ¿cómo crees que actuarías después?**

Me haría otro autotest antes de tomar cualquier decisión

Iría a un centro para que me hicieran otra prueba distinta para confirmar ese resultado positivo

Trataría de olvidarme del tema y no hacer nada más

No sé lo que haría en una situación así. No sé cómo reaccionaría

**¿En qué lugar te harías esa otra prueba?**

En un centro de salud

En un centro de planificación familiar

Laboratorio privado

Una farmacia

Centro de enfermedades de transmisión sexual o de diagnóstico del VIH/sida

Servicios médicos de empresa o centro de trabajo

ONGs de sida o drogas (en su sede o en unidad móvil)

Otro sitio. Especificar cuál:

**If you perform a self-test and the result happened to be positive ¿How do you think you would act?**

I would perform an additional self-test before making any decision

I would go to a center to take a confirmation test

I would try to forget about it

I am not sure what would I do.

**Where would you take the confirmation test?**

A primary care center

A family planning service

A private laboratory

A pharmacy

An anonymous medical center for the diagnosis of HIV and other STIs

A medical service at work

An NGO (at their office or in a mobile unit)

Others: please specify

**How would you prefer to use a self-test**

**Y ¿Cómo preferirías hacértelo?**

Solo

Acompañado de alguién

**How would you prefer to use it?**

Alone

Accompanied by someone

**Type of self-test preferred**

**Estas pruebas se pueden hacer con saliva o con dos gotitas de sangre del dedo, como la prueba del azúcar en diabéticos. Si tuvieras que hacerte el autotest ¿Qué preferirías utilizar?**

Sangre

Saliva

Me da lo mismo

**Self-tests can be performed either with saliva or with finger prick blood (similar to blood tests for diabetes). If you had to perform a self-test, which one would you use?**

Blood

Saliva

Either way

**Frequency of use if self-test kits were already available**

**Imagina que el auto test empezara ya a venderse en las farmacias. ¿Cuál de las siguientes frases resume mejor la forma en que lo emplearías?**

Yo no lo utilizaría nunca, iría siempre a que me la hicieran los profesionales

Sólo lo utilizaría alguna que otra vez, la mayoría de las veces iría a que me hicieran la prueba

Sería la forma habitual de realizármela, aunque alguna vez iría a que me la hicieran

Sería la forma habitual de realizármela y no creo que fuera a que me la hicieran, salvo que el autotest me diera positivo

**Imagine that self-testing kits began to be marketed in pharmacies right now, Which of the following statements would represent the use you would make of them?**

I would never use it, I would always leave testing in the hands of a professional

I would only use it occasionally, I would normally go somewhere to get tested

It would be my usual way of testing, although I would also go somewhere to get tested

It would be my usual way of testing, I do not think I would go somewhere else to get tested unless I got a positive self-test
