# Supplementary material for: HIV self-testing in Spain: A valuable testing option for men-who-have-sex-with-men who have never tested for HIV
Source: PLoS One. 2019 Feb 13;14(2):e0210637. doi: 10.1371/journal.pone.0210637 (PMC6373894; doi:10.1371/journal.pone.0210637)
Supplement: S1 Table — (DOCX) [file pone.0210637.s002.docx]

|  | **High intentions (N=2161)** | | **Medium/low intentions (N=428)** | | **Total (N=2589)** | | **Pearson chi-square test** |
| --- | --- | --- | --- | --- | --- | --- | --- |
|  | **N** | **%** | **N** | **%** |  |  | **p** |
|  |  |  |  |  |  |  |  |
| **Age** |  |  |  |  |  |  |  |
| Missing | 3 | 0.1 | 0 | 0.0 | 3 | 0.1 | (N/A) |
| **Place of residence** |  |  |  |  |  |  |  |
| Missing | 142 | 6.6 | 40 | 9.3 | 182 | 7.0 | 0.040 |
| **Settlement size (inhabitants)** |  |  |  |  |  |  |  |
| Missing | 157 | 7,3 | 41 | 9,6 | 198 | 7.6 | 0.100 |
| **Cohabitation** |  |  |  |  |  |  |  |
| Missing | 128 | 5,9 | 39 | 9,1 | 167 | 6.5 | 0,014 |
| **Disclosure of sexual orientation** |  |  |  |  |  |  |  |
| Missing | 119 | 5,5 | 41 | 9,6 | 160 | 6.2 | 0.001 |
| **Realation with gay culture** |  |  |  |  |  |  |  |
| Missing | 188 | 8.7 | 57 | 13.3 | 245 | 9.5 | 0.003 |
| **Anal intercourse & condom use** |  |  |  |  |  |  |  |
| Missing | 126 | 5.8 | 40 | 9.3 | 166 | 6.4 | 0.007 |
| **History from other STIs** |  |  |  |  |  |  |  |
| Missing | 147 | 6.8 | 41 | 9.6 | 188 | 7.3 | 0.043 |
| **Main reason for not testing for HIV** |  |  |  |  |  |  |  |
| Missing | 26 | 1.2 | 1 | 0.2 | 27 | 1.0 | 0.112* |
